# Supplementary material for: Functional brain architecture is associated with the rate of tau accumulation in Alzheimer’s disease
Source: Nat Commun. 2020 Jan 17;11:347. doi: 10.1038/s41467-019-14159-1 (PMC6969065; doi:10.1038/s41467-019-14159-1)
Supplement: Supplementary file 1 — Supplementary Information [file 41467_2019_14159_MOESM1_ESM.pdf]

Functional brain architecture is associated with the rate of tau accumulation in Alzheimer's disease

Franzmeier et al., Supplementary Figures:

Supplementary Figure 1: Brain parcellation and resting-state fMRI.

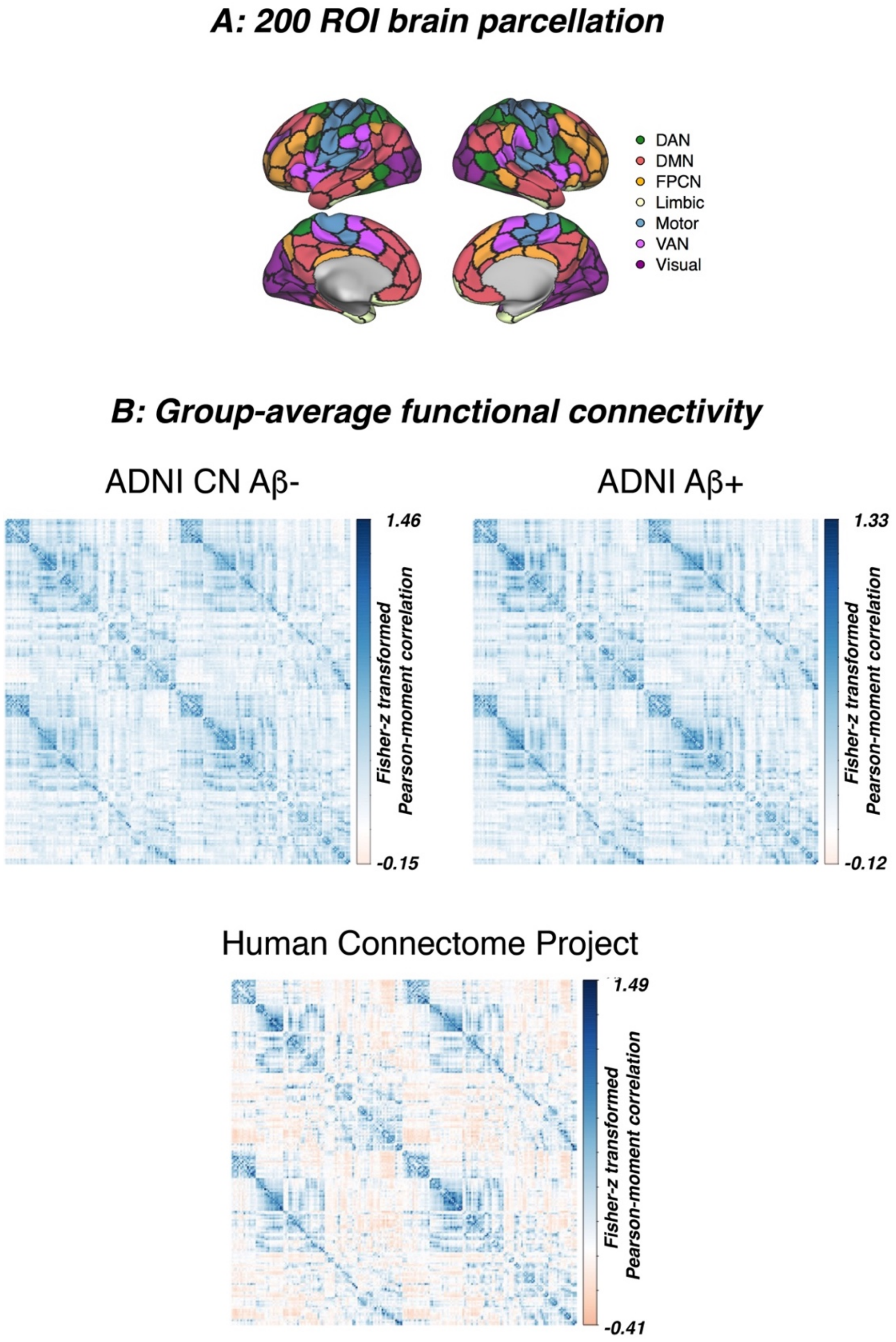

(A) Surface rendering of the 200 ROI brain parcellation that was applied to tau-PET and resting-state fMRI data for ROI based analyses. (B) Group-average functional connectivity matrices for 28 CN Aβ- and 53 Aβ+ of the ADNI sample, as well as for 500 subjects from the human connectome project (HCP). In ADNI, no Bonferroni-corrected differences ($p<0.05$) in

functional connectivity were found between the Aβ+ and CN-Aβ- in ANCOVAS controlling for age, sex, education and diagnosis.

Supplementary Figure 2: Covariance in tau-PET change.

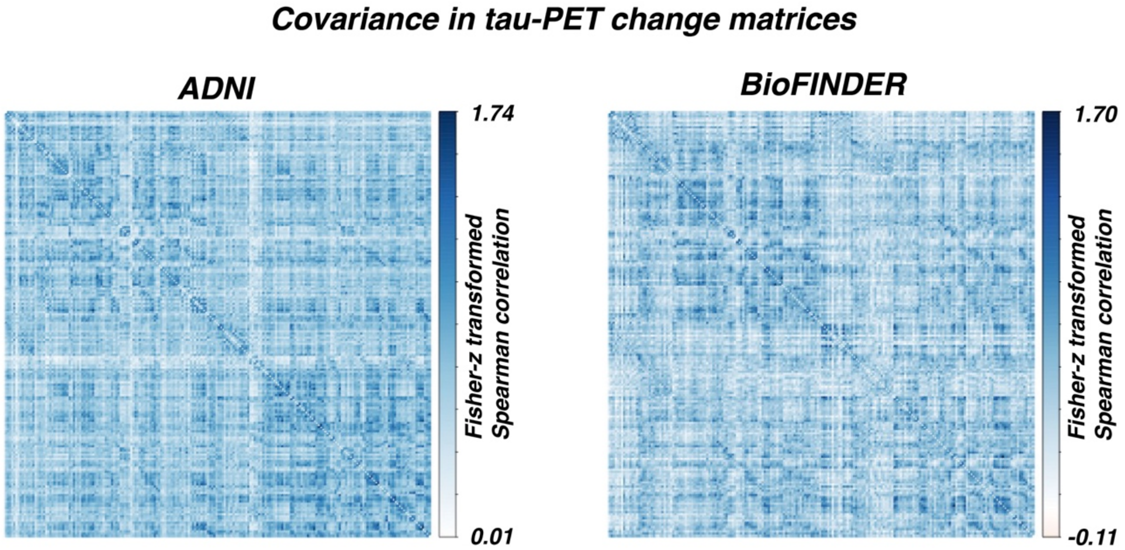

200x200 Covariance in tau-PET change matrices for the Aβ+ subjects of the ADNI (N=53, left panel) and BioFINDER (N=41, right panel) sample.

Supplementary Figure 3: Association between functional connectivity and covariance in tau-PET change.

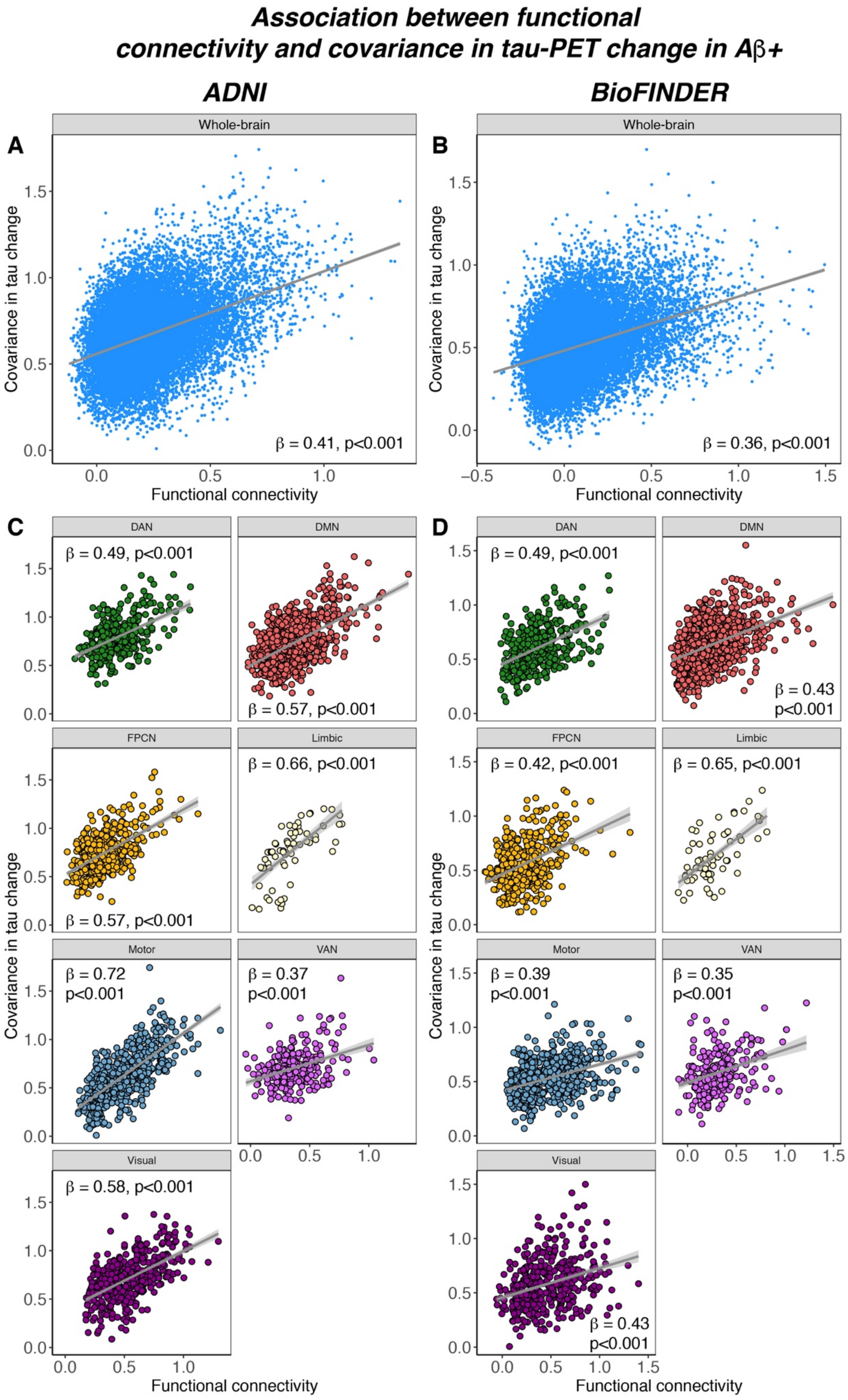

Scatterplots illustrating the association between group-average functional connectivity and covariance in tau-PET change in the Aβ+ groups of the ADNI (N=53) and BioFINDER (N=41) samples, for the whole brain (A&B) or for the 7 canonical brain networks (C&D) using the 200 ROI atlas. Standardized β- and p-values were derived from linear regression.

Supplementary Figure 4: Associations between functional connectivity and tau-PET change.

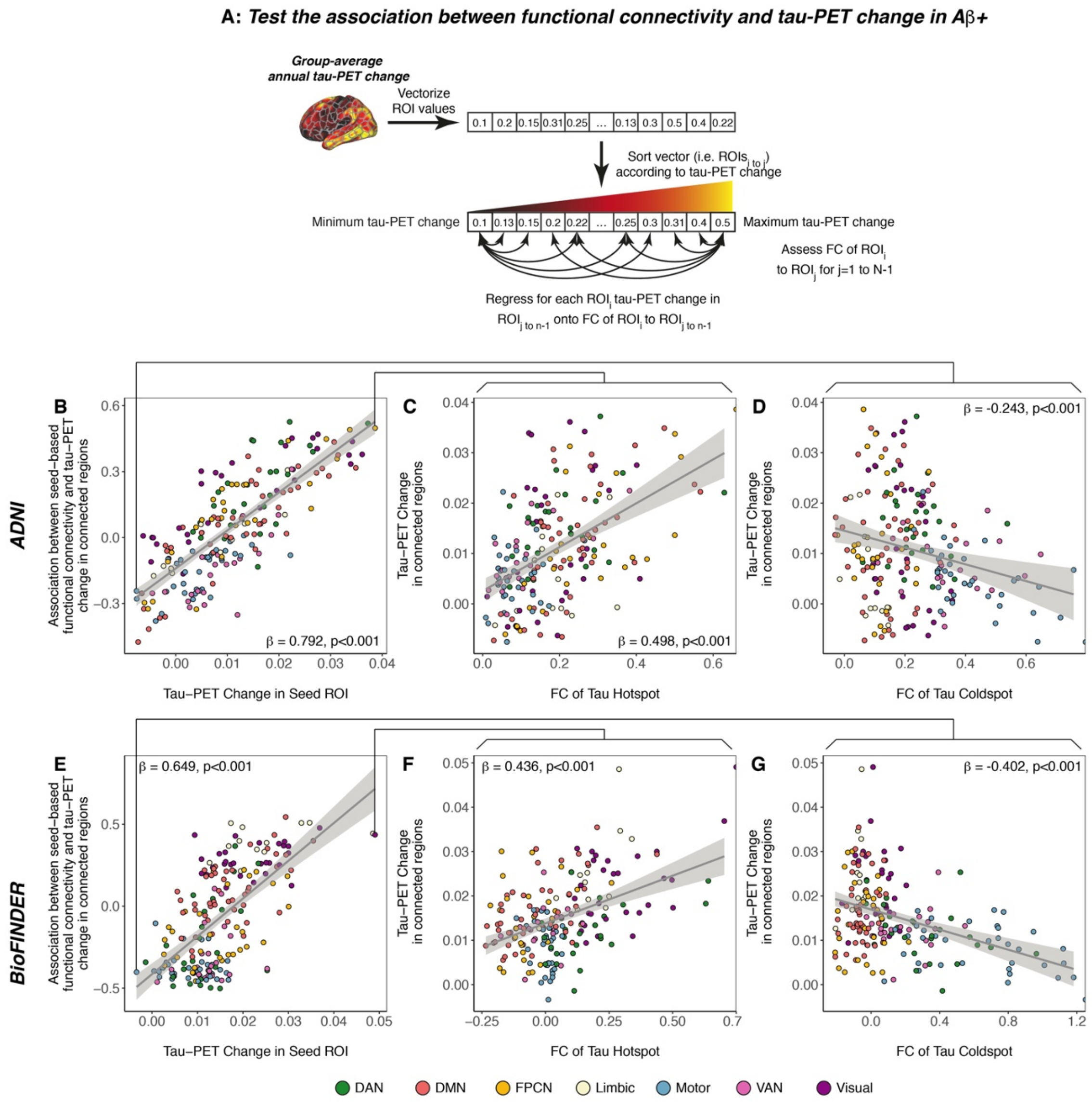

(A) Pipeline for testing the association between group-average functional connectivity and annual tau-PET change in the 53 Aβ+ from ADNI and 41 Aβ+ subjects from BioFINDER. Subjects using the 200ROI parcellation. For both ADNI (B) and BioFINDER (E), we plotted the association between annual tau-PET change of a seed-ROI (x-axis) and the regression derived association between its' functional connectivity to target regions and tau-PET change in the respective target regions (y-axis). Positive y-values indicate that higher FC to target regions is associated with higher annual tau-PET change, while negative y-values indicate that higher FC to target regions is associated with lower annual tau-PET changes. Illustration of the association between seed-based functional connectivity (x-axis) and annual tau-PET change in connected regions (y-axis) for ROIs with maximum (ADNI: C; BioFINDER: F) and minimum (ADNI: D; BioFINDER: G) annual tau-PET change. Source data are provided in a Source data file.

Supplementary Figure 5: Prediction of longitudinal tau-PET change.

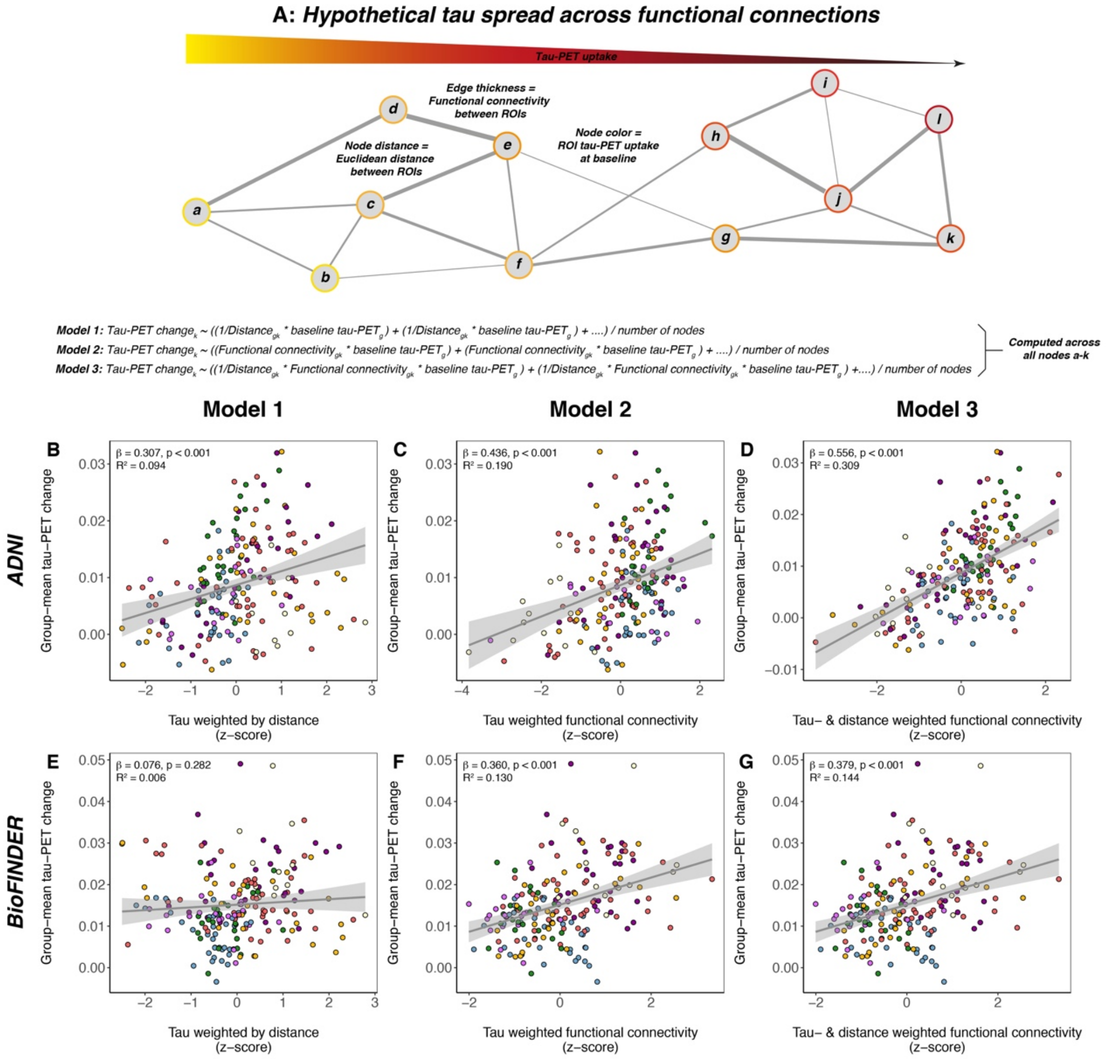

(A) Hypothetical network spreading model of tau pathology. Each node within the network represents a brain region, where color indicates local tau pathology, distance between regions indicates connection length (i.e. Euclidean distance) and edge thickness indicates functional connectivity strength. Example formulas for models 1-3 illustrate how we computed tau-weighted distance (Model 1), tau-weighted functional connectivity (Model 2) or tau- & distance-weighted functional connectivity (Model 3) that were used to model group-mean annual tau-PET change in the 53 Aβ+ ADNI (B-D) and 41 Aβ+ BioFINDER subjects (E-G) using the 200 ROI parcellation
