# Supplementary material for: Functional brain architecture is associated with the rate of tau accumulation in Alzheimer’s disease
Source: Nat Commun. 2020 Jan 17;11:347. doi: 10.1038/s41467-019-14159-1 (PMC6969065; doi:10.1038/s41467-019-14159-1)
Supplement: Supplementary file 3 — Description of Additional Supplementary Files [file 41467_2019_14159_MOESM3_ESM.pdf]

Description of Additional Supplementary Files

File Name: Supplementary Code 1

Description: Example R-Markdown code to simulate connectivity-related tau spreading analyses
