# Supplementary material for: Functional brain architecture is associated with the rate of tau accumulation in Alzheimer’s disease
Source: Nat Commun. 2020 Jan 17;11:347. doi: 10.1038/s41467-019-14159-1 (PMC6969065; doi:10.1038/s41467-019-14159-1)
Supplement: Supplementary file 4 — Supplementary Code 1 [file 41467_2019_14159_MOESM4_ESM.zip › 5.html]

Tau\_spreading\_Simulation.utf8.md


### TAU SPREADING ANALYSIS

# Tested with R-Studio - Versions 1.1.414 & 1.2.1578

### Prepare simulated data for tau vs. functional connectivity analysis

```
####---> 1. set to folder in which all files are stored ####
# (i.e. Tau_spreading_Simulation.R, Schaefer2018_200Parcels_7Networks_order_numeric.txt,
# Schaefer2018_400Parcels_7Networks_order_numeric.txt )

simulation_dir="/Volumes/Users/nfranzme/OneDrive/ISD/Manuscripts/Tau_Spread_longitudinal/Submission_NatComms/Revision2/Supplementary/Tau_spreading_Uploaded_software"

####---> 2. input meta data ####
# determine range of the atlas (e.g. ROIs range from 0 to 400)
Atlas_range=c(0,400)
Atlas_max=max(Atlas_range)
# determine the number of subjects
nsubs=53
# determine the number of volumes for a resting-state fMRI image
nvols_rsfMRI=200

####---> 3. simulate demographic data ####
dems=data.frame(Age=rnorm(nsubs, 60,5), Sex=sample(0:1, nsubs, replace=T), APOE=sample(0:2, nsubs, replace=T))

####---> 4. simulate baseline tau PET and annual tau PET changes ####
pet_annual_change_concat <- replicate(Atlas_max, rnorm(nsubs, mean=1,sd=1))
pet_mean_baseline <- replicate(Atlas_max, rnorm(nsubs, mean=1,sd=1))

####---> 5. create mean tau covariance matrix for simulated subjects ####
Tau_change_covariance <- psych::fisherz(cor(pet_annual_change_concat, method="spearman"))

corrplot::corrplot(Tau_change_covariance, diag = FALSE, 
                   tl.pos = "n", tl.cex = 0.5, method = "color", is.corr = FALSE)
```

```
####---> 6. create mean tau covariance matrix corrected for age, gender and ApoE ####
Tau_change_covariance_corrected=matrix(nrow=dim(pet_annual_change_concat)[2], ncol=dim(pet_annual_change_concat)[2])

for (i in 1:dim(pet_annual_change_concat)[2]){
  for (j in i:dim(pet_annual_change_concat)[2]){
    if (i!=j){
      irow=pet_annual_change_concat[,i]
      jrow=pet_annual_change_concat[,j]
      
      tmp=summary(lm.beta::lm.beta(lm(irow~jrow + dems$Age + dems$Sex + dems$APOE)))
      current_beta=tmp$coefficients["jrow", "Standardized"]
      Tau_change_covariance_corrected[i,j]=current_beta
      Tau_change_covariance_corrected[j,i]=current_beta
    }
    if (i==j){Tau_change_covariance_corrected[i,j]=NA}
  }
}

corrplot::corrplot(Tau_change_covariance_corrected, diag = FALSE, 
                   tl.pos = "n", tl.cex = 0.5, method = "color", is.corr = FALSE)
```

```
####---> 7. simulate subject-specific functional connectivity data ####
all_corrmats=array(rep(0, Atlas_max*Atlas_max*nsubs), dim=c(Atlas_max,Atlas_max, nsubs))
for (i in 1:nsubs){
  fMRI_timecourses=replicate(Atlas_max, rnorm(nvols_rsfMRI, mean=1,sd=1))
  corrmat_fisherz=psych::fisherz(cor(fMRI_timecourses, method="spearman"))
  all_corrmats[,,i]=corrmat_fisherz
}

####---> 8. create group-mean functional connectivity matrix  ####
mean_corrmat=apply(all_corrmats, c(1,2), mean)

corrplot::corrplot(mean_corrmat, diag = FALSE, 
                   tl.pos = "n", tl.cex = 0.5, method = "color", is.corr = FALSE)
```

```
####---> 9. simulate distance matrix ####
Schaefer_Euclidean_Distance_matrix <- replicate(Atlas_max, rnorm(Atlas_max, mean=1,sd=1))

corrplot::corrplot(Schaefer_Euclidean_Distance_matrix, diag = FALSE, 
                   tl.pos = "n", tl.cex = 0.5, method = "color", is.corr = FALSE)
```

### The following code assesses the association between functional connectivity and covariance in tau change

Step 1 tests the association between FC and covariance in tau change for the whole brain   
Step 2 tests the same association in a network specific manner (i.e. here a community structure file is required)   
Step 3 is analogous to step 1, where the covariance in tau change matrix is corrected for covariates   
Step 4 repeats step 1 by randomly drawing bootstrapped samples from the overall sample, based on which the covariance in tau change and functional connectivity matrices are assessed   
Step 5 restricts Step 1 to regions with high tau at baseline in steps of 25%

```
### ----> 1. Test the association between FC vs covariance in tau change: Whole brain ####
FC_linear=mean_corrmat[!is.infinite(mean_corrmat)]
TAU_linear=Tau_change_covariance[!is.infinite(mean_corrmat)]
Schaefer_Euclidean_Distance_matrix_linear=Schaefer_Euclidean_Distance_matrix[!is.infinite(mean_corrmat)]

df1=data.frame(FC=FC_linear, Tau=TAU_linear, Euclid=Schaefer_Euclidean_Distance_matrix_linear)

tmp <- lm.beta(lm(Tau~FC, data=df1)); summary(tmp)
```

```
## 
## Call:
## lm(formula = Tau ~ FC, data = df1)
## 
## Residuals:
##      Min       1Q   Median       3Q      Max 
## -0.72501 -0.09442 -0.00027  0.09419  0.68801 
## 
## Coefficients:
##               Estimate Standardized Std. Error t value Pr(>|t|)  
## (Intercept) -0.0006250    0.0000000  0.0003534  -1.768    0.077 .
## FC           0.0456613    0.0031527  0.0362536   1.259    0.208  
## ---
## Signif. codes:  0 '***' 0.001 '**' 0.01 '*' 0.05 '.' 0.1 ' ' 1
## 
## Residual standard error: 0.1412 on 159598 degrees of freedom
## Multiple R-squared:  9.939e-06,  Adjusted R-squared:  3.674e-06 
## F-statistic: 1.586 on 1 and 159598 DF,  p-value: 0.2079
```

```
tmp <- lm.beta(lm(Tau~Euclid, data=df1)); summary(tmp)
```

```
## 
## Call:
## lm(formula = Tau ~ Euclid, data = df1)
## 
## Residuals:
##      Min       1Q   Median       3Q      Max 
## -0.72475 -0.09449 -0.00021  0.09411  0.68912 
## 
## Coefficients:
##               Estimate Standardized Std. Error t value Pr(>|t|)
## (Intercept) -0.0002102    0.0000000  0.0004995  -0.421    0.674
## Euclid      -0.0004122   -0.0029254  0.0003527  -1.169    0.243
## 
## Residual standard error: 0.1412 on 159598 degrees of freedom
## Multiple R-squared:  8.558e-06,  Adjusted R-squared:  2.292e-06 
## F-statistic: 1.366 on 1 and 159598 DF,  p-value: 0.2425
```

```
tmp <- lm.beta(lm(Tau~FC + Euclid, data=df1)); summary(tmp)
```

```
## 
## Call:
## lm(formula = Tau ~ FC + Euclid, data = df1)
## 
## Residuals:
##      Min       1Q   Median       3Q      Max 
## -0.72515 -0.09443 -0.00025  0.09416  0.68878 
## 
## Coefficients:
##               Estimate Standardized Std. Error t value Pr(>|t|)
## (Intercept) -0.0002131    0.0000000  0.0004995  -0.427    0.670
## FC           0.0456019    0.0031486  0.0362536   1.258    0.208
## Euclid      -0.0004116   -0.0029210  0.0003527  -1.167    0.243
## 
## Residual standard error: 0.1412 on 159597 degrees of freedom
## Multiple R-squared:  1.847e-05,  Adjusted R-squared:  5.94e-06 
## F-statistic: 1.474 on 2 and 159597 DF,  p-value: 0.229
```

```
p1 <- ggplot(data=df1, aes(x=FC, y=Tau)) +
  xlab("Functional connectivity") + ylab("Covariance in tau change") + theme_bw() + theme(
    plot.title = element_text(size=14, face="bold",hjust = 0.5),
    axis.title.x = element_text(size=14),
    axis.title.y = element_text(size=14), axis.text=element_text(size=14))+ theme(panel.grid.major = element_blank(),  panel.grid.minor = element_blank(), axis.line = element_line(colour = "black"))
p1 <- p1 + geom_point(size=0.5, colour="dodgerblue") + geom_smooth(method=lm, se=TRUE, colour="gray56") 
p1
```

```
### ----> 2. Test the association between FC vs covariance in tau change: Network-specific ####
# read in community structure
Community <- read.table(paste0(simulation_dir, "/Schaefer2018_",Atlas_max,"Parcels_7Networks_order_numeric.txt"))
Community$names <- mapvalues(Community$V1, from=c(1,2,3,4,5,6,7), to=c("Visual", "Motor", "DAN", "VAN", "Limbic", "FPCN", "DMN"))

# plot network-specific associations
Networks=c("Visual", "Motor", "DAN", "VAN", "Limbic", "FPCN", "DMN")
n=0
for (i in 1:length(Networks)){
  n=n+1
  current_Network=Networks[i]
  current_network_elements=which(Community$names==current_Network)
  current_FC_network=mean_corrmat[current_network_elements, current_network_elements]
  current_FC_linear=current_FC_network[!is.infinite(current_FC_network)]
  Tau_network=Tau_change_covariance[current_network_elements, current_network_elements]
  TAU_linear=Tau_network[!is.infinite(current_FC_network)]
  
  df_network_FC_tau_covariance <- data.frame(FC=current_FC_linear, TAU=TAU_linear)
  df_network_FC_tau_covariance$Network=current_Network
  
  if(n==1){df_network_FC_tau_covariance_concat=df_network_FC_tau_covariance}
  if(n>1){df_network_FC_tau_covariance_concat=rbind(df_network_FC_tau_covariance_concat, df_network_FC_tau_covariance)}
  
}

p_network_specific <- ggplot(df_network_FC_tau_covariance_concat, aes(x=FC, y=TAU)) + geom_point(aes(fill=Network), colour="black",pch=21, size=2) + xlab("Functional connectivity") + ylab("Covariance in tau change") + 
  scale_fill_manual(values=c("forestgreen" ,"indianred2" ,"darkgoldenrod1" ,"lemonchiffon1" ,"skyblue3" ,"mediumorchid1" ,"magenta4"))  + geom_smooth(method=lm, se=TRUE, colour="gray56") + theme_bw()  + theme(
    plot.title = element_text(size=14, face="bold",hjust = 0.5),
    axis.title.x = element_text(size=14),
    axis.title.y = element_text(size=14), axis.text=element_text(size=14))+ theme(panel.grid.major = element_blank(),  panel.grid.minor = element_blank(), axis.line = element_line(colour = "black"), legend.position = "none") +
  facet_wrap(.~Network, ncol=2) 
p_network_specific
```

```
# Visual
current_network_elements=which(Community$names=="Visual")
FC_network=mean_corrmat[current_network_elements, current_network_elements]
FC_linear=FC_network[!is.infinite(FC_network)]

Tau_network=Tau_change_covariance[current_network_elements, current_network_elements]
TAU_linear=Tau_network[!is.infinite(FC_network)]

tmp <- lm.beta(lm(TAU_linear~FC_linear)); summary(tmp)
```

```
## 
## Call:
## lm(formula = TAU_linear ~ FC_linear)
## 
## Residuals:
##      Min       1Q   Median       3Q      Max 
## -0.47125 -0.09307 -0.00584  0.08994  0.51836 
## 
## Coefficients:
##               Estimate Standardized Std. Error t value Pr(>|t|)
## (Intercept)  0.0002093    0.0000000  0.0023029   0.091    0.928
## FC_linear   -0.3307813   -0.0228727  0.2390500  -1.384    0.167
## 
## Residual standard error: 0.1393 on 3658 degrees of freedom
## Multiple R-squared:  0.0005232,  Adjusted R-squared:  0.0002499 
## F-statistic: 1.915 on 1 and 3658 DF,  p-value: 0.1665
```

```
# Motor
current_network_elements=which(Community$names=="Motor")
FC_network=mean_corrmat[current_network_elements, current_network_elements]
FC_linear=FC_network[!is.infinite(FC_network)]

Tau_network=Tau_change_covariance[current_network_elements, current_network_elements]
TAU_linear=Tau_network[!is.infinite(FC_network)]

tmp <- lm.beta(lm(TAU_linear~FC_linear)); summary(tmp)
```

```
## 
## Call:
## lm(formula = TAU_linear ~ FC_linear)
## 
## Residuals:
##      Min       1Q   Median       3Q      Max 
## -0.48317 -0.09017 -0.00035  0.09242  0.54490 
## 
## Coefficients:
##              Estimate Standardized Std. Error t value Pr(>|t|)
## (Intercept) -0.001532     0.000000   0.001854  -0.826    0.409
## FC_linear    0.180951     0.012388   0.190968   0.948    0.343
## 
## Residual standard error: 0.1418 on 5850 degrees of freedom
## Multiple R-squared:  0.0001535,  Adjusted R-squared:  -1.746e-05 
## F-statistic: 0.8978 on 1 and 5850 DF,  p-value: 0.3434
```

```
# DAN
current_network_elements=which(Community$names=="DAN")
FC_network=mean_corrmat[current_network_elements, current_network_elements]
FC_linear=FC_network[!is.infinite(FC_network)]

Tau_network=Tau_change_covariance[current_network_elements, current_network_elements]
TAU_linear=Tau_network[!is.infinite(FC_network)]

tmp <- lm.beta(lm(TAU_linear~FC_linear)); summary(tmp)
```

```
## 
## Call:
## lm(formula = TAU_linear ~ FC_linear)
## 
## Residuals:
##      Min       1Q   Median       3Q      Max 
## -0.48188 -0.09414 -0.00064  0.09378  0.47336 
## 
## Coefficients:
##              Estimate Standardized Std. Error t value Pr(>|t|)
## (Intercept)  0.004061     0.000000   0.003179   1.277    0.202
## FC_linear   -0.185496    -0.012424   0.328288  -0.565    0.572
## 
## Residual standard error: 0.1446 on 2068 degrees of freedom
## Multiple R-squared:  0.0001544,  Adjusted R-squared:  -0.0003291 
## F-statistic: 0.3193 on 1 and 2068 DF,  p-value: 0.5721
```

```
# VAN
current_network_elements=which(Community$names=="VAN")
FC_network=mean_corrmat[current_network_elements, current_network_elements]
FC_linear=FC_network[!is.infinite(FC_network)]

Tau_network=Tau_change_covariance[current_network_elements, current_network_elements]
TAU_linear=Tau_network[!is.infinite(FC_network)]

tmp <- lm.beta(lm(TAU_linear~FC_linear)); summary(tmp)
```

```
## 
## Call:
## lm(formula = TAU_linear ~ FC_linear)
## 
## Residuals:
##      Min       1Q   Median       3Q      Max 
## -0.60049 -0.09387  0.00081  0.09875  0.40400 
## 
## Coefficients:
##              Estimate Standardized Std. Error t value Pr(>|t|)
## (Intercept) -0.001202     0.000000   0.003065  -0.392    0.695
## FC_linear    0.481416     0.032684   0.316760   1.520    0.129
## 
## Residual standard error: 0.1425 on 2160 degrees of freedom
## Multiple R-squared:  0.001068,   Adjusted R-squared:  0.0006058 
## F-statistic:  2.31 on 1 and 2160 DF,  p-value: 0.1287
```

```
# Limbic
current_network_elements=which(Community$names=="Limbic")
FC_network=mean_corrmat[current_network_elements, current_network_elements]
FC_linear=FC_network[!is.infinite(FC_network)]

Tau_network=Tau_change_covariance[current_network_elements, current_network_elements]
TAU_linear=Tau_network[!is.infinite(FC_network)]

tmp <- lm.beta(lm(TAU_linear~FC_linear)); summary(tmp)
```

```
## 
## Call:
## lm(formula = TAU_linear ~ FC_linear)
## 
## Residuals:
##      Min       1Q   Median       3Q      Max 
## -0.47200 -0.09677  0.00460  0.10212  0.51267 
## 
## Coefficients:
##             Estimate Standardized Std. Error t value Pr(>|t|)  
## (Intercept) 0.002002     0.000000   0.005826   0.344   0.7312  
## FC_linear   1.328224     0.085988   0.604556   2.197   0.0284 *
## ---
## Signif. codes:  0 '***' 0.001 '**' 0.01 '*' 0.05 '.' 0.1 ' ' 1
## 
## Residual standard error: 0.1484 on 648 degrees of freedom
## Multiple R-squared:  0.007394,   Adjusted R-squared:  0.005862 
## F-statistic: 4.827 on 1 and 648 DF,  p-value: 0.02837
```

```
# FPCN
current_network_elements=which(Community$names=="FPCN")
FC_network=mean_corrmat[current_network_elements, current_network_elements]
FC_linear=FC_network[!is.infinite(FC_network)]

Tau_network=Tau_change_covariance[current_network_elements, current_network_elements]
TAU_linear=Tau_network[!is.infinite(FC_network)]

tmp <- lm.beta(lm(TAU_linear~FC_linear)); summary(tmp)
```

```
## 
## Call:
## lm(formula = TAU_linear ~ FC_linear)
## 
## Residuals:
##      Min       1Q   Median       3Q      Max 
## -0.66758 -0.09356 -0.00628  0.09031  0.51535 
## 
## Coefficients:
##             Estimate Standardized Std. Error t value Pr(>|t|)
## (Intercept) 0.003646     0.000000   0.002775   1.314    0.189
## FC_linear   0.376511     0.024767   0.295225   1.275    0.202
## 
## Residual standard error: 0.1429 on 2650 degrees of freedom
## Multiple R-squared:  0.0006134,  Adjusted R-squared:  0.0002363 
## F-statistic: 1.626 on 1 and 2650 DF,  p-value: 0.2023
```

```
# DMN
current_network_elements=which(Community$names=="DMN")
FC_network=mean_corrmat[current_network_elements, current_network_elements]
FC_linear=FC_network[!is.infinite(FC_network)]

Tau_network=Tau_change_covariance[current_network_elements, current_network_elements]
TAU_linear=Tau_network[!is.infinite(FC_network)]

tmp <- lm.beta(lm(TAU_linear~FC_linear)); summary(tmp)
```

```
## 
## Call:
## lm(formula = TAU_linear ~ FC_linear)
## 
## Residuals:
##      Min       1Q   Median       3Q      Max 
## -0.47006 -0.09566 -0.00075  0.09342  0.49277 
## 
## Coefficients:
##               Estimate Standardized Std. Error t value Pr(>|t|)  
## (Intercept) -0.0005806    0.0000000  0.0015724  -0.369   0.7119  
## FC_linear    0.2738432    0.0189973  0.1592729   1.719   0.0856 .
## ---
## Signif. codes:  0 '***' 0.001 '**' 0.01 '*' 0.05 '.' 0.1 ' ' 1
## 
## Residual standard error: 0.1423 on 8188 degrees of freedom
## Multiple R-squared:  0.0003609,  Adjusted R-squared:  0.0002388 
## F-statistic: 2.956 on 1 and 8188 DF,  p-value: 0.08559
```

```
### ----> 3. Test the association between FC vs covariance in tau change: Whole brain - corrected for covariates ####
FC_linear=mean_corrmat[!is.infinite(mean_corrmat)]
TAU_linear=Tau_change_covariance_corrected[!is.infinite(mean_corrmat)]
Schaefer_Euclidean_Distance_matrix_linear=Schaefer_Euclidean_Distance_matrix[!is.infinite(mean_corrmat)]

df1=data.frame(FC=FC_linear, Tau=TAU_linear, Euclid=Schaefer_Euclidean_Distance_matrix_linear)
tmp <- lm.beta(lm(TAU_linear~FC_linear)); summary(tmp)
```

```
## 
## Call:
## lm(formula = TAU_linear ~ FC_linear)
## 
## Residuals:
##      Min       1Q   Median       3Q      Max 
## -0.58288 -0.09754  0.00014  0.09724  0.58432 
## 
## Coefficients:
##               Estimate Standardized Std. Error t value Pr(>|t|)  
## (Intercept) -0.0008527    0.0000000  0.0003569  -2.389   0.0169 *
## FC_linear    0.0244795    0.0016735  0.0366146   0.669   0.5038  
## ---
## Signif. codes:  0 '***' 0.001 '**' 0.01 '*' 0.05 '.' 0.1 ' ' 1
## 
## Residual standard error: 0.1426 on 159598 degrees of freedom
## Multiple R-squared:  2.801e-06,  Adjusted R-squared:  -3.465e-06 
## F-statistic: 0.447 on 1 and 159598 DF,  p-value: 0.5038
```

```
### ----> 4. Test the association between FC vs covariance in tau change: Whole brain - bootstrapped ####
bootstrapping_iterations=100
corrmats_tmp=all_corrmats
pet_change_tmp=pet_annual_change_concat
Schaefer_Euclidean_Distance_matrix_linear=Schaefer_Euclidean_Distance_matrix[!is.infinite(mean_corrmat)]

Tau_change_covariance_FC_bootstrapped_correlations=numeric()
Tau_change_covariance_Distance_bootstrapped_correlations=numeric()
for (bootrep in 1:bootstrapping_iterations){
  
  # determine bootstap ids  
  sample_pool=c(1:dim(corrmats_tmp)[3])
  sampled_ids=as.numeric(replicate(1,sample(sample_pool, replace=TRUE)))
  
  # select bootstrapped FC
  corrmats_boot=corrmats_tmp[,,sampled_ids]
  corrmats_boot_mean=apply(corrmats_boot, c(1,2), mean)
  
  # select bootstrapped tau change
  pet_change_boot=pet_change_tmp[sampled_ids, ]
  
  # assess bootstrapped tau covariance matrix
  TAU_change_covariance_boot=fisherz(cor(pet_change_boot, method=c("spearman")))
  
  # vectorize data
  FC_linear_boot=corrmats_boot_mean[!is.infinite(corrmats_boot_mean)]
  TAU_change_covariance_linear_boot=TAU_change_covariance_boot[!is.infinite(TAU_change_covariance_boot)]
  
  
  tmp <- summary(lm.beta(lm(TAU_change_covariance_linear_boot~FC_linear_boot)))
  
  
  Tau_change_covariance_FC_bootstrapped_correlations[bootrep]=tmp$coefficients["FC_linear_boot","Standardized"]
  
}

# test whether beta-value distribution is different from zero
t.test(Tau_change_covariance_FC_bootstrapped_correlations)
```

```
## 
##  One Sample t-test
## 
## data:  Tau_change_covariance_FC_bootstrapped_correlations
## t = 3.9473, df = 99, p-value = 0.000148
## alternative hypothesis: true mean is not equal to 0
## 95 percent confidence interval:
##  0.0005974179 0.0018051385
## sample estimates:
##   mean of x 
## 0.001201278
```

```
# create density plot of beta values with confidence intervals
x <- Tau_change_covariance_FC_bootstrapped_correlations
d <- as.data.frame(x=x)

p <- ggplot(data = d) + theme_bw() + 
  geom_density(aes(x=x, y = ..density..), color = 'black')
# new code is below
q5 <- quantile(x,.05)
q95 <- quantile(x,.95)
medx <- median(x)
x.dens <- density(x)
df.dens <- data.frame(x = x.dens$x, y = x.dens$y)
density_bootstrapping=p + geom_area(data = subset(df.dens, x >= q5 & x <= q95), 
                                    aes(x=x,y=y), fill = 'coral', alpha=0.5) + xlab("Association between functional connectivity\nand covariance in tau change\n(bootstrapped b-values)") + theme_bw() + theme(
                                      plot.title = element_text(size=14, face="bold",hjust = 0.5),
                                      axis.title.x = element_text(size=14),
                                      axis.title.y = element_text(size=14), axis.text=element_text(size=14))+ theme(panel.grid.major = element_blank(),  panel.grid.minor = element_blank(), axis.line = element_line(colour = "black"))
density_bootstrapping
```

```
### ----> 5. Test the association between FC vs covariance in tau change: Whole brain - restrict to regions with 75%/50%/25% highest tau at baseline ####

# 75% highest tau regions
tmp_pet_mean <- apply(pet_mean_baseline, 2, mean)
tmp_pet_mean_75p <- tmp_pet_mean>quantile(tmp_pet_mean)[4]
mean_corrmat_thr=mean_corrmat[tmp_pet_mean_75p==1, tmp_pet_mean_75p==1]
FC_linear_thr=mean_corrmat_thr[!is.infinite(mean_corrmat_thr)]
Tau_change_covariance_thr=Tau_change_covariance[tmp_pet_mean_75p==1, tmp_pet_mean_75p==1]
TAU_linear_thr=Tau_change_covariance_thr[!is.infinite(Tau_change_covariance_thr)]

tmp <- lm.beta(lm(TAU_linear_thr~FC_linear_thr)); summary(tmp)
```

```
## 
## Call:
## lm(formula = TAU_linear_thr ~ FC_linear_thr)
## 
## Residuals:
##      Min       1Q   Median       3Q      Max 
## -0.66571 -0.09175  0.00011  0.09225  0.51517 
## 
## Coefficients:
##                Estimate Standardized Std. Error t value Pr(>|t|)
## (Intercept)    0.001664     0.000000   0.001392   1.195    0.232
## FC_linear_thr -0.077823    -0.005515   0.141840  -0.549    0.583
## 
## Residual standard error: 0.1385 on 9898 degrees of freedom
## Multiple R-squared:  3.041e-05,  Adjusted R-squared:  -7.061e-05 
## F-statistic: 0.301 on 1 and 9898 DF,  p-value: 0.5832
```

```
# 50% highest tau regions
tmp_pet_mean <- apply(pet_mean_baseline, 2, mean)
tmp_pet_mean_50p <- tmp_pet_mean>quantile(tmp_pet_mean)[3]
mean_corrmat_thr=mean_corrmat[tmp_pet_mean_50p==1, tmp_pet_mean_50p==1]
FC_linear_thr=mean_corrmat_thr[!is.infinite(mean_corrmat_thr)]
Tau_change_covariance_thr=Tau_change_covariance[tmp_pet_mean_50p==1, tmp_pet_mean_50p==1]
TAU_linear_thr=Tau_change_covariance_thr[!is.infinite(Tau_change_covariance_thr)]

tmp <- lm.beta(lm(TAU_linear_thr~FC_linear_thr)); summary(tmp)
```

```
## 
## Call:
## lm(formula = TAU_linear_thr ~ FC_linear_thr)
## 
## Residuals:
##      Min       1Q   Median       3Q      Max 
## -0.66387 -0.09355 -0.00005  0.09347  0.52132 
## 
## Coefficients:
##                 Estimate Standardized Std. Error t value Pr(>|t|)
## (Intercept)   -0.0001529    0.0000000  0.0007039  -0.217    0.828
## FC_linear_thr  0.0073299    0.0005126  0.0716737   0.102    0.919
## 
## Residual standard error: 0.1404 on 39798 degrees of freedom
## Multiple R-squared:  2.628e-07,  Adjusted R-squared:  -2.486e-05 
## F-statistic: 0.01046 on 1 and 39798 DF,  p-value: 0.9185
```

```
# 25% highest tau regions
tmp_pet_mean <- apply(pet_mean_baseline, 2, mean)
tmp_pet_mean_25p <- tmp_pet_mean>quantile(tmp_pet_mean)[2]
mean_corrmat_thr=mean_corrmat[tmp_pet_mean_25p==1, tmp_pet_mean_25p==1]
FC_linear_thr=mean_corrmat_thr[!is.infinite(mean_corrmat_thr)]
Tau_change_covariance_thr=Tau_change_covariance[tmp_pet_mean_25p==1, tmp_pet_mean_25p==1]
TAU_linear_thr=Tau_change_covariance_thr[!is.infinite(Tau_change_covariance_thr)]

tmp <- lm.beta(lm(TAU_linear_thr~FC_linear_thr)); summary(tmp)
```

```
## 
## Call:
## lm(formula = TAU_linear_thr ~ FC_linear_thr)
## 
## Residuals:
##      Min       1Q   Median       3Q      Max 
## -0.66312 -0.09434 -0.00024  0.09452  0.58381 
## 
## Coefficients:
##                 Estimate Standardized Std. Error t value Pr(>|t|)  
## (Intercept)   -0.0008982    0.0000000  0.0004704  -1.910   0.0562 .
## FC_linear_thr  0.0142225    0.0009865  0.0481383   0.295   0.7676  
## ---
## Signif. codes:  0 '***' 0.001 '**' 0.01 '*' 0.05 '.' 0.1 ' ' 1
## 
## Residual standard error: 0.1409 on 89698 degrees of freedom
## Multiple R-squared:  9.732e-07,  Adjusted R-squared:  -1.018e-05 
## F-statistic: 0.08729 on 1 and 89698 DF,  p-value: 0.7676
```

### The following code tests whether functional connectivity of regions with high/low tau change predicts similar rates of tau change in connected regions

Step 1 tests for example regions with highest/lowest tau change, whether higher connectivity predicts higher/lower tau change in connected regions   
Step 2 tests across all brain regions, whether higher connectivity predicts higher/lower tau change in connected regions   
Step 3 repeats step 1 by randomly drawing bootstrapped samples from the overall sample, based on which the annual tau change and functional connectivity are assessed

```
FC_tmp=mean_corrmat
pet_annual_change_mean_tmp <- colMeans(pet_annual_change_concat)

### ----> 1. Test whether connectivity of the hihg/low tau change regions predicts tau change in connected regions: example regions ####

# Region with maximum tau change 
max_tau <- which.max(pet_annual_change_mean_tmp); max_tau=max_tau[[1]]
max_tau_FC <- FC_tmp[1:Atlas_max,max_tau]

df=data.frame(pet_annual_change_mean_tmp, max_tau_FC, Community$names)
names(df)[3]="Network"
tmp <- lm.beta(lm(max_tau_FC[!is.infinite(max_tau_FC)]~pet_annual_change_mean_tmp[!is.infinite(max_tau_FC)])); summary(tmp)
```

```
## 
## Call:
## lm(formula = max_tau_FC[!is.infinite(max_tau_FC)] ~ pet_annual_change_mean_tmp[!is.infinite(max_tau_FC)])
## 
## Residuals:
##        Min         1Q     Median         3Q        Max 
## -0.0269593 -0.0064531  0.0006314  0.0070502  0.0302122 
## 
## Coefficients:
##                                                       Estimate
## (Intercept)                                          -0.004067
## pet_annual_change_mean_tmp[!is.infinite(max_tau_FC)]  0.004467
##                                                      Standardized
## (Intercept)                                              0.000000
## pet_annual_change_mean_tmp[!is.infinite(max_tau_FC)]     0.064491
##                                                      Std. Error t value
## (Intercept)                                            0.003469  -1.172
## pet_annual_change_mean_tmp[!is.infinite(max_tau_FC)]   0.003469   1.288
##                                                      Pr(>|t|)
## (Intercept)                                             0.242
## pet_annual_change_mean_tmp[!is.infinite(max_tau_FC)]    0.199
## 
## Residual standard error: 0.009922 on 397 degrees of freedom
## Multiple R-squared:  0.004159,   Adjusted R-squared:  0.001651 
## F-statistic: 1.658 on 1 and 397 DF,  p-value: 0.1986
```

```
p <-   ggplot(data=df, aes(x=max_tau_FC, y=pet_annual_change_mean_tmp)) + geom_point(aes(fill=Network), colour="black",pch=21, size=2) + xlab("\nFC") + ylab("Tau-PET Change\n in connected regions\n") + geom_smooth(method=lm, se=TRUE, colour="gray56") + theme_bw() + scale_fill_manual(values=c("forestgreen" ,"indianred2" ,"darkgoldenrod1" ,"lemonchiffon1" ,"skyblue3" ,"mediumorchid1" ,"magenta4")) +scale_shape_manual(values=c(21,22,24,23))+ theme(
  plot.title = element_text(size=14, face="bold",hjust = 0.5),
  axis.title.x = element_text(size=14),
  axis.title.y = element_text(size=14), axis.text=element_text(size=14))+ theme(panel.grid.major = element_blank(),  panel.grid.minor = element_blank(), axis.line = element_line(colour = "black"), legend.position="none")
p
```

```
## Warning: Removed 1 rows containing non-finite values (stat_smooth).
```

```
# Region with minimum tau change 
min_tau <- which.min(pet_annual_change_mean_tmp); min_tau=min_tau[[1]]
min_tau_FC <- FC_tmp[1:Atlas_max,min_tau]

df=data.frame(pet_annual_change_mean_tmp, min_tau_FC, Community$names)
names(df)[3]="Network"
tmp <- lm.beta(lm(min_tau_FC[!is.infinite(min_tau_FC)]~pet_annual_change_mean_tmp[!is.infinite(min_tau_FC)])); summary(tmp)
```

```
## 
## Call:
## lm(formula = min_tau_FC[!is.infinite(min_tau_FC)] ~ pet_annual_change_mean_tmp[!is.infinite(min_tau_FC)])
## 
## Residuals:
##       Min        1Q    Median        3Q       Max 
## -0.033678 -0.006446  0.000125  0.006065  0.025086 
## 
## Coefficients:
##                                                        Estimate
## (Intercept)                                          -0.0004274
## pet_annual_change_mean_tmp[!is.infinite(min_tau_FC)]  0.0004701
##                                                      Standardized
## (Intercept)                                             0.0000000
## pet_annual_change_mean_tmp[!is.infinite(min_tau_FC)]    0.0069265
##                                                      Std. Error t value
## (Intercept)                                           0.0034139  -0.125
## pet_annual_change_mean_tmp[!is.infinite(min_tau_FC)]  0.0034061   0.138
##                                                      Pr(>|t|)
## (Intercept)                                              0.90
## pet_annual_change_mean_tmp[!is.infinite(min_tau_FC)]     0.89
## 
## Residual standard error: 0.009738 on 397 degrees of freedom
## Multiple R-squared:  4.798e-05,  Adjusted R-squared:  -0.002471 
## F-statistic: 0.01905 on 1 and 397 DF,  p-value: 0.8903
```

```
p <-   ggplot(data=df, aes(x=min_tau_FC, y=pet_annual_change_mean_tmp)) + geom_point(aes(fill=Network), colour="black",pch=21, size=2) + xlab("\nFC") + ylab("Tau-PET Change\n in connected regions\n") + geom_smooth(method=lm, se=TRUE, colour="gray56") + theme_bw() + scale_fill_manual(values=c("forestgreen" ,"indianred2" ,"darkgoldenrod1" ,"lemonchiffon1" ,"skyblue3" ,"mediumorchid1" ,"magenta4")) +scale_shape_manual(values=c(21,22,24,23))+ theme(
  plot.title = element_text(size=14, face="bold",hjust = 0.5),
  axis.title.x = element_text(size=14),
  axis.title.y = element_text(size=14), axis.text=element_text(size=14))+ theme(panel.grid.major = element_blank(),  panel.grid.minor = element_blank(), axis.line = element_line(colour = "black"), legend.position="none")
p
```

```
## Warning: Removed 1 rows containing non-finite values (stat_smooth).
```

```
### ----> 2. Test whether connectivity of the maximum/minimum tau change regions predicts tau change in connected regions: rank ROIs across tau range ####
rank_tau <- rank(pet_annual_change_mean_tmp)
rank_results = data.frame(matrix(vector(), Atlas_max,6, dimnames=list(c(), c("R", "P", "Rank", "ROI","Tau", "Network"))), stringsAsFactors=F)

for (j in 1:Atlas_max){
  
  current_ROI <- j
  current_FC <- FC_tmp[1:Atlas_max,current_ROI]
  linear_model <- summary(lm.beta(lm(pet_annual_change_mean_tmp[!is.infinite(current_FC)]~current_FC[!is.infinite(current_FC)])))
  
  rank_results[j,1]=linear_model$coefficients["current_FC[!is.infinite(current_FC)]", "Standardized"]
  rank_results[j,2]=linear_model$coefficients["current_FC[!is.infinite(current_FC)]", "Pr(>|t|)"]
  rank_results[j,3]=rank_tau[j]
  rank_results[j,5]=pet_annual_change_mean_tmp[[j]]
  rank_results[j,6]=Community$names[j]
}

tmp <- lm.beta(lm(rank_results$R~rank_results$Tau)); summary(tmp)
```

```
## 
## Call:
## lm(formula = rank_results$R ~ rank_results$Tau)
## 
## Residuals:
##       Min        1Q    Median        3Q       Max 
## -0.175541 -0.032480  0.000108  0.032217  0.157848 
## 
## Coefficients:
##                   Estimate Standardized Std. Error t value Pr(>|t|)
## (Intercept)      6.419e-03    0.000e+00  1.790e-02   0.359    0.720
## rank_results$Tau 8.108e-05    2.274e-04  1.788e-02   0.005    0.996
## 
## Residual standard error: 0.05172 on 398 degrees of freedom
## Multiple R-squared:  5.17e-08,   Adjusted R-squared:  -0.002513 
## F-statistic: 2.058e-05 on 1 and 398 DF,  p-value: 0.9964
```

```
p <- ggplot(data=rank_results, aes(x=Tau, y=R)) + geom_point(aes(fill=Network), colour="black",pch=21, size=2) + xlab("\nTau-PET Change in Seed ROI") + ylab("Correlation between Seed FC\n and Tau-PET Change in connected regions\n")  + geom_smooth(method=lm, se=TRUE, colour="gray56") + theme_bw() + scale_fill_manual(values=c("forestgreen" ,"indianred2" ,"darkgoldenrod1" ,"lemonchiffon1" ,"skyblue3" ,"mediumorchid1" ,"magenta4"))  + theme(
  plot.title = element_text(size=14, face="bold",hjust = 0.5),
  axis.title.x = element_text(size=14),
  axis.title.y = element_text(size=14), axis.text=element_text(size=14))+ theme(panel.grid.major = element_blank(),  panel.grid.minor = element_blank(), axis.line = element_line(colour = "black"), legend.position = "none") 
p
```

```
### ----> 3. Test whether connectivity of the maximum/minimum tau change regions predicts tau change in connected regions: rank ROIs across tau range - bootstrapped ####

bootstrapping_iterations=100
corrmats_tmp=all_corrmats
pet_change_tmp=pet_annual_change_concat
hotcoldchange_bootstrapped_correlations=numeric()

for (bootrep in 1:bootstrapping_iterations){
  
  # determine bootstap ids  
  sample_pool=c(1:dim(corrmats_tmp)[3])
  sampled_ids=as.numeric(replicate(1,sample(sample_pool, replace=TRUE)))
  
  # select bootstrapped FC
  corrmats_boot=corrmats_tmp[,,sampled_ids]
  corrmats_boot_mean=apply(corrmats_boot, c(1,2), mean)
  
  # select bootstrapped tau change
  pet_change_boot=colMeans(pet_change_tmp[sampled_ids, ])
  FC_tmp=corrmats_boot_mean
  
  # rank order
  rank_tau <- rank(pet_change_boot)
  rank_results = data.frame(matrix(vector(), Atlas_max,6, dimnames=list(c(), c("R", "P", "Rank", "ROI","Tau", "Network"))), stringsAsFactors=F)
  
  for (j in 1:Atlas_max){
    current_ROI <- j
    current_FC <- FC_tmp[1:Atlas_max,current_ROI]
    linear_model <- summary(lm.beta(lm(pet_change_boot[!is.infinite(current_FC)]~current_FC[!is.infinite(current_FC)])))
    rank_results[j,1]=linear_model$coefficients["current_FC[!is.infinite(current_FC)]", "Standardized"]
    rank_results[j,2]=linear_model$coefficients["current_FC[!is.infinite(current_FC)]", "Pr(>|t|)"]
    rank_results[j,3]=rank_tau[j]
    rank_results[j,5]=pet_change_boot[[j]]
    rank_results[j,6]=Community$names[j]
    
  }
  
  hotcoldchange_bootstrapped_correlations[bootrep]=rcorr(rank_results$R, rank_results$Tau, type=c("pearson"))[[1]][1,2]
  
}

# test whether beta-value distribution is different from zero
t.test(hotcoldchange_bootstrapped_correlations)
```

```
## 
##  One Sample t-test
## 
## data:  hotcoldchange_bootstrapped_correlations
## t = -0.99119, df = 99, p-value = 0.324
## alternative hypothesis: true mean is not equal to 0
## 95 percent confidence interval:
##  -0.020440399  0.006821865
## sample estimates:
##    mean of x 
## -0.006809267
```

```
# create density plot of beta values with confidence intervals
x <- hotcoldchange_bootstrapped_correlations
d <- as.data.frame(x=x)

p <- ggplot(data = d) + theme_bw() + 
  geom_density(aes(x=x, y = ..density..), color = 'black', adjust=1)
# new code is below
q5 <- quantile(x,.05)
q95 <- quantile(x,.95)
medx <- median(x)
x.dens <- density(x)
df.dens <- data.frame(x = x.dens$x, y = x.dens$y)
density_bootstrapping_hot_coldchange=p + geom_area(data = subset(df.dens, x >= q5 & x <= q95), 
                                                   aes(x=x,y=y), fill = 'coral', alpha=0.5) + xlab("Association between seed FC\nand tau-PET change in connected regions\n(bootstrapped b-values)") + theme_bw() + theme(
                                                     plot.title = element_text(size=14, face="bold",hjust = 0.5),
                                                     axis.title.x = element_text(size=14),
                                                     axis.title.y = element_text(size=14), axis.text=element_text(size=14))+ theme(panel.grid.major = element_blank(),  panel.grid.minor = element_blank(), axis.line = element_line(colour = "black"))
density_bootstrapping_hot_coldchange
```

### The following code assesses three tau spreading models that take into account tau at baseline, functional connectivity and distance between regions

Model 1 uses baseline tau levels weighted by distance (i.e. distance-weighted tau)   
Model 2 uses baseline tau levels weighted by functional connectivity (i.e. connectivity-weighted tau)   
Model 3 uses baseline tau levels weighted by functional connectivity and distance (i.e. connectivity & distance-weighted tau)

Step 1 assesses the accuracy of all models on the group level   
Step 2 repeats step 1, by randomly drawing bootstrapped samples from the overall sample, based on which group-average tau at baseline, tau change and functional connectivity are determined   
Step 3 assesses the accuracy of all models on the subject level   
Step 4 assesses whether prediction accuracy on the subject level is influenced by age, gender and ApoE

```
### ----> 1. Test 3 model approaches at the group level ####

# Select group-average FC matrix and remove diagonal
corrmats_tmp=mean_corrmat
corrmats_tmp[corrmats_tmp==Inf] = NA

# Select PET means and change rates
pet_mean_early=colMeans(pet_mean_baseline)
pet_mean_change=colMeans(pet_annual_change_concat)

# weight FC by tau and/or distance
Tau_weighted_by_distance=t(t(Schaefer_Euclidean_Distance_matrix) * I(pet_mean_early))
FC_weighted_by_tau=t(t(corrmats_tmp) * I(pet_mean_early))
FC_weighted_by_distance=corrmats_tmp*(Schaefer_Euclidean_Distance_matrix)
FC_weighted_by_distance_and_tau=t(t(FC_weighted_by_distance) * I(pet_mean_early))

# compute input weights
Input_weights_tau_weighted_by_distance=rowMeans(Tau_weighted_by_distance, na.rm=T)
Input_weights_FC_weighted_by_tau=rowMeans(FC_weighted_by_tau, na.rm=T)
Input_weights_FC_weighted_by_distance_and_tau=rowMeans(FC_weighted_by_distance_and_tau, na.rm=T)

# check associations between input weights and tau change
# Model 1: tau weighted by distance
df=data.frame(Input_weights_tau_weighted_by_distance, pet_mean_change, pet_mean_early, Network=Community$names)
tmp <- lm.beta(lm(pet_mean_change~Input_weights_tau_weighted_by_distance, data=df)); summary(tmp)
```

```
## 
## Call:
## lm(formula = pet_mean_change ~ Input_weights_tau_weighted_by_distance, 
##     data = df)
## 
## Residuals:
##      Min       1Q   Median       3Q      Max 
## -0.44323 -0.09489 -0.00553  0.10238  0.43623 
## 
## Coefficients:
##                                         Estimate Standardized Std. Error
## (Intercept)                             0.995022     0.000000   0.148266
## Input_weights_tau_weighted_by_distance -0.004087    -0.001397   0.146672
##                                        t value Pr(>|t|)    
## (Intercept)                              6.711 6.67e-11 ***
## Input_weights_tau_weighted_by_distance  -0.028    0.978    
## ---
## Signif. codes:  0 '***' 0.001 '**' 0.01 '*' 0.05 '.' 0.1 ' ' 1
## 
## Residual standard error: 0.145 on 398 degrees of freedom
## Multiple R-squared:  1.951e-06,  Adjusted R-squared:  -0.002511 
## F-statistic: 0.0007764 on 1 and 398 DF,  p-value: 0.9778
```

```
p <- ggplot(data=df, aes(x=scale(Input_weights_tau_weighted_by_distance), y=pet_mean_change)) + geom_point(aes(fill=Network), colour="black",pch=21, size=2) + xlab("\nTau weighted by distance") + ylab("Tau-PET change\n") + geom_smooth(method=lm, se=TRUE, colour="gray56") + theme_bw() + scale_fill_manual(values=c("forestgreen" ,"indianred2" ,"darkgoldenrod1" ,"lemonchiffon1" ,"skyblue3" ,"mediumorchid1" ,"magenta4"))  + theme(
  plot.title = element_text(size=14, face="bold",hjust = 0.5),
  axis.title.x = element_text(size=14),
  axis.title.y = element_text(size=14), axis.text=element_text(size=14))+ theme(panel.grid.major = element_blank(),  panel.grid.minor = element_blank(), axis.line = element_line(colour = "black"), legend.position = "none") 
p
```

```
# Model 2: tau weighted FC
df=data.frame(Input_weights_FC_weighted_by_tau, pet_mean_change, pet_mean_early, Network=Community$names)
tmp <- lm.beta(lm(pet_mean_change~Input_weights_FC_weighted_by_tau, data=df)); summary(tmp)
```

```
## 
## Call:
## lm(formula = pet_mean_change ~ Input_weights_FC_weighted_by_tau, 
##     data = df)
## 
## Residuals:
##      Min       1Q   Median       3Q      Max 
## -0.44698 -0.09771  0.00068  0.10411  0.42838 
## 
## Coefficients:
##                                   Estimate Standardized Std. Error t value
## (Intercept)                       0.989291     0.000000   0.007222 136.982
## Input_weights_FC_weighted_by_tau 35.743102     0.125230  14.194180   2.518
##                                  Pr(>|t|)    
## (Intercept)                        <2e-16 ***
## Input_weights_FC_weighted_by_tau   0.0122 *  
## ---
## Signif. codes:  0 '***' 0.001 '**' 0.01 '*' 0.05 '.' 0.1 ' ' 1
## 
## Residual standard error: 0.1439 on 398 degrees of freedom
## Multiple R-squared:  0.01568,    Adjusted R-squared:  0.01321 
## F-statistic: 6.341 on 1 and 398 DF,  p-value: 0.01219
```

```
p <- ggplot(data=df, aes(x=scale(Input_weights_FC_weighted_by_tau), y=pet_mean_change)) + geom_point(aes(fill=Network), colour="black",pch=21, size=2) + xlab("\nTau weighted by functional connectivity") + ylab("Tau-PET change\n") + geom_smooth(method=lm, se=TRUE, colour="gray56") + theme_bw() + scale_fill_manual(values=c("forestgreen" ,"indianred2" ,"darkgoldenrod1" ,"lemonchiffon1" ,"skyblue3" ,"mediumorchid1" ,"magenta4"))  + theme(
  plot.title = element_text(size=14, face="bold",hjust = 0.5),
  axis.title.x = element_text(size=14),
  axis.title.y = element_text(size=14), axis.text=element_text(size=14))+ theme(panel.grid.major = element_blank(),  panel.grid.minor = element_blank(), axis.line = element_line(colour = "black"), legend.position = "none") 
p
```

```
# Model 3: FC weighted by Tau and Distance
df=data.frame(Input_weights_FC_weighted_by_distance_and_tau, pet_mean_change, pet_mean_early, Network=Community$names)
tmp <- lm.beta(lm(pet_mean_change~Input_weights_FC_weighted_by_distance_and_tau, data=df)); summary(tmp)
```

```
## 
## Call:
## lm(formula = pet_mean_change ~ Input_weights_FC_weighted_by_distance_and_tau, 
##     data = df)
## 
## Residuals:
##      Min       1Q   Median       3Q      Max 
## -0.44755 -0.09676 -0.00072  0.10148  0.45721 
## 
## Coefficients:
##                                                Estimate Standardized
## (Intercept)                                    0.990177     0.000000
## Input_weights_FC_weighted_by_distance_and_tau 25.125869     0.130850
##                                               Std. Error t value Pr(>|t|)
## (Intercept)                                     0.007194 137.642  < 2e-16
## Input_weights_FC_weighted_by_distance_and_tau   9.542347   2.633  0.00879
##                                                  
## (Intercept)                                   ***
## Input_weights_FC_weighted_by_distance_and_tau ** 
## ---
## Signif. codes:  0 '***' 0.001 '**' 0.01 '*' 0.05 '.' 0.1 ' ' 1
## 
## Residual standard error: 0.1438 on 398 degrees of freedom
## Multiple R-squared:  0.01712,    Adjusted R-squared:  0.01465 
## F-statistic: 6.933 on 1 and 398 DF,  p-value: 0.008791
```

```
p <- ggplot(data=df, aes(x=scale(Input_weights_FC_weighted_by_distance_and_tau), y=pet_mean_change)) + geom_point(aes(fill=Network), colour="black",pch=21, size=2) + xlab("\nTau weighted by distance and functional connectivity") + ylab("Tau-PET change\n") + geom_smooth(method=lm, se=TRUE, colour="gray56") + theme_bw() + scale_fill_manual(values=c("forestgreen" ,"indianred2" ,"darkgoldenrod1" ,"lemonchiffon1" ,"skyblue3" ,"mediumorchid1" ,"magenta4"))  + theme(
  plot.title = element_text(size=14, face="bold",hjust = 0.5),
  axis.title.x = element_text(size=14),
  axis.title.y = element_text(size=14), axis.text=element_text(size=14))+ theme(panel.grid.major = element_blank(),  panel.grid.minor = element_blank(), axis.line = element_line(colour = "black"), legend.position = "none") 
p
```

```
### ----> 2. Test 3 model approaches at the group level - bootstrapped ####

# determine input data
bootstrapping_iterations=100
corrmats_tmp=all_corrmats
pet_change_tmp=pet_annual_change_concat
pet_early_tmp=pet_mean_baseline

# determine output data
Summary_Input_weights_tau_weighted_by_distance=numeric()
Summary_Input_weights_FC_weighted_by_tau=numeric()
Summary_Input_weights_FC_weighted_by_distance_and_tau=numeric()

for (bootrep in 1:bootstrapping_iterations){

  # determine bootstap ids  
  sample_pool=c(1:dim(corrmats_tmp)[3])
  sampled_ids=as.numeric(replicate(1,sample(sample_pool, replace=TRUE)))
  
  # extract bootstrapped data
  corrmats_boot=apply(corrmats_tmp[,,sampled_ids], c(1,2), mean)
  pet_change_boot=apply(pet_change_tmp[sampled_ids,], 2, median)
  pet_early_boot=apply(pet_early_tmp[sampled_ids,], 2, median)
  
  # Select FC matrix of Abpos and remove diagonal
  corrmats_boot[corrmats_boot==Inf] = NA
  
  # weight FC by tau and/or distance
  Tau_weighted_by_distance=t(t(Schaefer_Euclidean_Distance_matrix) * I(pet_early_boot))
  FC_weighted_by_tau=t(t(corrmats_boot) * I(pet_early_boot))
  FC_weighted_by_distance_and_tau=t(t(FC_weighted_by_distance) * I(pet_early_boot))
  
  # compute input weights
  Input_weights_tau_weighted_by_distance=rowMeans(Tau_weighted_by_distance, na.rm=T)
  Input_weights_FC_weighted_by_tau=rowMeans(FC_weighted_by_tau, na.rm=T)
  Input_weights_FC_weighted_by_distance_and_tau=rowMeans(FC_weighted_by_distance_and_tau, na.rm=T)
  
  g0=c(1:Atlas_max)
  # check associations between FC and tau change
  tmp <- summary(lm.beta(lm(pet_mean_change[g0]~Input_weights_FC_weighted_by_tau[g0])))
  Summary_Input_weights_FC_weighted_by_tau[bootrep]=tmp$coefficients["Input_weights_FC_weighted_by_tau[g0]", "Standardized"]
  
  tmp <- summary(lm.beta(lm(pet_mean_change[g0]~Input_weights_tau_weighted_by_distance[g0])))
  Summary_Input_weights_tau_weighted_by_distance[bootrep]=tmp$coefficients["Input_weights_tau_weighted_by_distance[g0]", "Standardized"]
  
  tmp <- summary(lm.beta(lm(pet_mean_change[g0]~Input_weights_FC_weighted_by_distance_and_tau[g0])))
  Summary_Input_weights_FC_weighted_by_distance_and_tau[bootrep]=tmp$coefficients["Input_weights_FC_weighted_by_distance_and_tau[g0]", "Standardized"]
}

df=data.frame(Iteration=1:bootrep, Summary_Input_weights_FC_weighted_by_tau,Summary_Input_weights_tau_weighted_by_distance,Summary_Input_weights_FC_weighted_by_distance_and_tau)
df_bootstrapping_summary=df

df=data.frame(input=c(rep("tau_by_dist", bootrep), rep("FC_by_tau", bootrep), rep("FC_by_tau_and_dist", bootrep)), correlation=c(Summary_Input_weights_tau_weighted_by_distance, Summary_Input_weights_FC_weighted_by_tau, Summary_Input_weights_FC_weighted_by_distance_and_tau))
df$input_factor=factor(df$input, levels=c("tau_by_dist", "FC_by_tau", "FC_by_tau_and_dist"))
p <- ggplot(df, aes(x=reorder(input, correlation, FUN = median), y=correlation, group=input_factor)) + geom_violin(alpha=0.2, fill="dodgerblue", width=0.7) + geom_boxplot(notch = T, alpha=1, width=0.4)+ xlab("") + ylab("Bootstrapped prediction (b-values) of tau-PET change\n") + geom_smooth(method=lm, se=TRUE, colour="gray56") + theme_bw()  + theme(
  plot.title = element_text(size=14, face="bold",hjust = 0.5),
  axis.title.x = element_text(size=14),
  axis.title.y = element_text(size=14), axis.text=element_text(size=14))+ theme(panel.grid.major = element_blank(),  panel.grid.minor = element_blank(), axis.line = element_line(colour = "black"), legend.position = "none") 
p
```

```
### ----> 3. Test 3 model approaches at the subject level ####

R_FC=numeric()
R_distance_tau=numeric()
R_distance_FC_tau=numeric()

for (i in 1:nsubs){
  
  # Select FC matrix of Abpos and remove diagonal & negatives
  corrmats_tmp=all_corrmats[,,i]
  corrmats_tmp[is.infinite(corrmats_tmp)]=NA
  # Select PET means and change rates
  pet_mean_early=pet_mean_baseline[i,]
  pet_mean_change=pet_annual_change_concat[i,]
  
  # weight FC by tau and/or distance
  FC_weighted_by_tau=t(t(corrmats_tmp) * I(pet_mean_early))
  Tau_weighted_by_distance=t(t(Schaefer_Euclidean_Distance_matrix) * I(pet_mean_early)); Tau_weighted_by_distance[is.infinite(Tau_weighted_by_distance)]=NA
  FC_weighted_by_distance_and_tau=t(t(FC_weighted_by_distance) * I(pet_mean_early))
  
  # compute input weights
  Input_weights_FC_weighted_by_tau=rowMeans(FC_weighted_by_tau, na.rm=T)
  Input_weights_tau_weighted_by_distance=rowMeans(Tau_weighted_by_distance, na.rm=T)
  Input_weights_FC_weighted_by_distance_and_tau=rowMeans(FC_weighted_by_distance_and_tau, na.rm=T)
  
  # check associations between input weights and tau change
  g0=c(1:Atlas_max)
  
  
  tmp <- summary(lm.beta(lm(pet_mean_change[g0]~Input_weights_FC_weighted_by_tau[g0])))
  R_FC[i]=tmp$coefficients["Input_weights_FC_weighted_by_tau[g0]", "Standardized"]
  
  tmp <- summary(lm.beta(lm(pet_mean_change[g0]~Input_weights_tau_weighted_by_distance[g0])))
  R_distance_tau[i]=tmp$coefficients["Input_weights_tau_weighted_by_distance[g0]", "Standardized"]
  
  tmp <- summary(lm.beta(lm(pet_mean_change[g0]~Input_weights_FC_weighted_by_distance_and_tau[g0])))
  R_distance_FC_tau[i]=tmp$coefficients["Input_weights_FC_weighted_by_distance_and_tau[g0]", "Standardized"]
  
}

tmp_df=data.frame(association=c(R_distance_tau, R_FC, R_distance_FC_tau), Group=c(rep("distance_tau", 53), rep("FC_tau", 53), rep("FC_distance_tau", 53)), mean_tau_change=rowMeans(pet_annual_change_concat))
tmp=(aov(association~Group, data=tmp_df)); summary(tmp); TukeyHSD(tmp)
```

```
##              Df Sum Sq  Mean Sq F value Pr(>F)
## Group         2 0.0065 0.003247    1.34  0.265
## Residuals   156 0.3780 0.002423
```

```
##   Tukey multiple comparisons of means
##     95% family-wise confidence level
## 
## Fit: aov(formula = association ~ Group, data = tmp_df)
## 
## $Group
##                                      diff          lwr         upr
## FC_distance_tau-distance_tau  0.014293111 -0.008334233 0.036920454
## FC_tau-distance_tau           0.001615301 -0.021012043 0.024242644
## FC_tau-FC_distance_tau       -0.012677810 -0.035305153 0.009949533
##                                  p adj
## FC_distance_tau-distance_tau 0.2962310
## FC_tau-distance_tau          0.9843934
## FC_tau-FC_distance_tau       0.3830244
```

```
describeBy(R_distance_tau); t.test(R_distance_tau)
```

```
## Warning in describeBy(R_distance_tau): no grouping variable requested
```

```
##    vars  n mean   sd median trimmed  mad   min  max range  skew kurtosis
## X1    1 53    0 0.05      0       0 0.05 -0.11 0.09  0.21 -0.24    -0.74
##      se
## X1 0.01
```

```
## 
##  One Sample t-test
## 
## data:  R_distance_tau
## t = -0.22367, df = 52, p-value = 0.8239
## alternative hypothesis: true mean is not equal to 0
## 95 percent confidence interval:
##  -0.01395885  0.01115904
## sample estimates:
##    mean of x 
## -0.001399906
```

```
describeBy(R_FC); t.test(R_FC)
```

```
## Warning in describeBy(R_FC): no grouping variable requested
```

```
##    vars  n mean   sd median trimmed  mad   min  max range  skew kurtosis
## X1    1 53    0 0.06      0       0 0.05 -0.13 0.13  0.26 -0.11    -0.35
##      se
## X1 0.01
```

```
## 
##  One Sample t-test
## 
## data:  R_FC
## t = 0.026943, df = 52, p-value = 0.9786
## alternative hypothesis: true mean is not equal to 0
## 95 percent confidence interval:
##  -0.01582641  0.01625720
## sample estimates:
##    mean of x 
## 0.0002153947
```

```
describeBy(R_distance_FC_tau); t.test(R_distance_FC_tau)
```

```
## Warning in describeBy(R_distance_FC_tau): no grouping variable requested
```

```
##    vars  n mean   sd median trimmed  mad   min  max range  skew kurtosis
## X1    1 53 0.01 0.04   0.01    0.01 0.04 -0.08 0.11  0.19 -0.01    -0.52
##      se
## X1 0.01
```

```
## 
##  One Sample t-test
## 
## data:  R_distance_FC_tau
## t = 2.2087, df = 52, p-value = 0.03163
## alternative hypothesis: true mean is not equal to 0
## 95 percent confidence interval:
##  0.001179283 0.024607126
## sample estimates:
## mean of x 
## 0.0128932
```

```
p <- ggplot(tmp_df, aes(x=reorder(Group, association, FUN = median), y=association, group=Group)) + geom_violin(alpha=0.2, fill="dodgerblue", width=0.7) + geom_boxplot(notch = T, alpha=1, width=0.4) + xlab("") + ylab("Subject-level prediction (b-values) of tau-PET change\n")  + theme_bw() + theme(
  plot.title = element_text(size=14, face="bold",hjust = 0.5),
  axis.title.x = element_text(size=14),
  axis.title.y = element_text(size=14), axis.text=element_text(size=14))+ theme(panel.grid.major = element_blank(),  panel.grid.minor = element_blank(), axis.line = element_line(colour = "black"), legend.position = "none") 
p
```

```
### ----> 4. Assess effects of age, gender and ApoE on subject-level prediction performance ####
subject_level_prediction_models <- dems
subject_level_prediction_models$Model1 <- subset(tmp_df, Group=="distance_tau")$association
subject_level_prediction_models$Model2 <- subset(tmp_df, Group=="FC_tau")$association
subject_level_prediction_models$Model3 <- subset(tmp_df, Group=="FC_distance_tau")$association

tmp <- lm(Model1~Age, data=subject_level_prediction_models); summary(tmp)
```

```
## 
## Call:
## lm(formula = Model1 ~ Age, data = subject_level_prediction_models)
## 
## Residuals:
##       Min        1Q    Median        3Q       Max 
## -0.109610 -0.035523  0.003225  0.036586  0.094061 
## 
## Coefficients:
##               Estimate Std. Error t value Pr(>|t|)
## (Intercept)  0.0282119  0.0798353   0.353    0.725
## Age         -0.0004944  0.0013288  -0.372    0.711
## 
## Residual standard error: 0.04595 on 51 degrees of freedom
## Multiple R-squared:  0.002707,   Adjusted R-squared:  -0.01685 
## F-statistic: 0.1384 on 1 and 51 DF,  p-value: 0.7114
```

```
tmp <- lm(Model2~Age, data=subject_level_prediction_models); summary(tmp)
```

```
## 
## Call:
## lm(formula = Model2 ~ Age, data = subject_level_prediction_models)
## 
## Residuals:
##      Min       1Q   Median       3Q      Max 
## -0.11567 -0.03242 -0.01431  0.03523  0.11810 
## 
## Coefficients:
##              Estimate Std. Error t value Pr(>|t|)  
## (Intercept) -0.168141   0.099337  -1.693   0.0966 .
## Age          0.002811   0.001653   1.700   0.0952 .
## ---
## Signif. codes:  0 '***' 0.001 '**' 0.01 '*' 0.05 '.' 0.1 ' ' 1
## 
## Residual standard error: 0.05717 on 51 degrees of freedom
## Multiple R-squared:  0.05363,    Adjusted R-squared:  0.03508 
## F-statistic:  2.89 on 1 and 51 DF,  p-value: 0.0952
```

```
tmp <- lm(Model3~Age, data=subject_level_prediction_models); summary(tmp)
```

```
## 
## Call:
## lm(formula = Model3 ~ Age, data = subject_level_prediction_models)
## 
## Residuals:
##       Min        1Q    Median        3Q       Max 
## -0.088334 -0.027455  0.002012  0.025765  0.093242 
## 
## Coefficients:
##               Estimate Std. Error t value Pr(>|t|)
## (Intercept) -0.0436534  0.0741403  -0.589    0.559
## Age          0.0009442  0.0012340   0.765    0.448
## 
## Residual standard error: 0.04267 on 51 degrees of freedom
## Multiple R-squared:  0.01135,    Adjusted R-squared:  -0.008038 
## F-statistic: 0.5854 on 1 and 51 DF,  p-value: 0.4477
```

```
tmp <- aov(Model1~Sex, data=subject_level_prediction_models); summary(tmp)
```

```
##             Df Sum Sq  Mean Sq F value Pr(>F)
## Sex          1 0.0033 0.003295   1.606  0.211
## Residuals   51 0.1047 0.002052
```

```
tmp <- aov(Model2~Sex, data=subject_level_prediction_models); summary(tmp)
```

```
##             Df  Sum Sq  Mean Sq F value Pr(>F)
## Sex          1 0.00415 0.004149    1.23  0.273
## Residuals   51 0.17199 0.003372
```

```
tmp <- aov(Model3~Sex, data=subject_level_prediction_models); summary(tmp)
```

```
##             Df  Sum Sq   Mean Sq F value Pr(>F)
## Sex          1 0.00023 0.0002345   0.128  0.722
## Residuals   51 0.09368 0.0018369
```

```
tmp <- aov(Model1~APOE, data=subject_level_prediction_models); summary(tmp)
```

```
##             Df Sum Sq   Mean Sq F value Pr(>F)
## APOE         1  0.000 0.0000036   0.002  0.967
## Residuals   51  0.108 0.0021167
```

```
tmp <- aov(Model2~APOE, data=subject_level_prediction_models); summary(tmp)
```

```
##             Df  Sum Sq  Mean Sq F value Pr(>F)
## APOE         1 0.00282 0.002818   0.829  0.367
## Residuals   51 0.17332 0.003398
```

```
tmp <- aov(Model3~APOE, data=subject_level_prediction_models); summary(tmp)
```

```
##             Df  Sum Sq  Mean Sq F value Pr(>F)
## APOE         1 0.00057 0.000569   0.311   0.58
## Residuals   51 0.09335 0.001830
```
