# Supplementary material for: Functional brain architecture is associated with the rate of tau accumulation in Alzheimer’s disease
Source: Nat Commun. 2020 Jan 17;11:347. doi: 10.1038/s41467-019-14159-1 (PMC6969065; doi:10.1038/s41467-019-14159-1)
Supplement: Supplementary file 5 — Reporting Summary [file 41467_2019_14159_MOESM5_ESM.pdf]

nature research

Corresponding author(s): Nicolai Franzmeier

Last updated by author(s): Oct 7th 2019

Reporting Summary

Nature Research wishes to improve the reproducibility of the work that we publish. This form provides structure for consistency and transparency in reporting. For further information on Nature Research policies, see Authors & Referees and the Editorial Policy Checklist.

Statistics

For all statistical analyses, confirm that the following items are present in the figure legend, table legend, main text, or Methods section.

n/a	Confirmed	
☐	☒	The exact sample size (*n*) for each experimental group/condition, given as a discrete number and unit of measurement
☐	☒	A statement on whether measurements were taken from distinct samples or whether the same sample was measured repeatedly
☐	☒	The statistical test(s) used AND whether they are one- or two-sided<br>*Only common tests should be described solely by name; describe more complex techniques in the Methods section.*
☐	☒	A description of all covariates tested
☒	☐	A description of any assumptions or corrections, such as tests of normality and adjustment for multiple comparisons
☐	☒	A full description of the statistical parameters including central tendency (e.g. means) or other basic estimates (e.g. regression coefficient) AND variation (e.g. standard deviation) or associated estimates of uncertainty (e.g. confidence intervals)
☐	☒	For null hypothesis testing, the test statistic (e.g. *F*, *t*, *r*) with confidence intervals, effect sizes, degrees of freedom and *P* value noted<br>*Give P values as exact values whenever suitable.*
☒	☐	For Bayesian analysis, information on the choice of priors and Markov chain Monte Carlo settings
☒	☐	For hierarchical and complex designs, identification of the appropriate level for tests and full reporting of outcomes
☐	☒	Estimates of effect sizes (e.g. Cohen's *d*, Pearson's *r*), indicating how they were calculated

Our web collection on statistics for biologists contains articles on many of the points above.

Software and code

Policy information about availability of computer code

Data collection	Data used in preparation of this manuscript were obtained from the ADNI database (adni. loni.usc.edu). As an independent validation sample, we included 57 participants from the BioFINDER cohort (http://biofinder.se)
Data analysis	All data was analyzed using RStudio statistical software, Version 1.1.414

For manuscripts utilizing custom algorithms or software that are central to the research but not yet described in published literature, software must be made available to editors/reviewers. We strongly encourage code deposition in a community repository (e.g. GitHub). See the Nature Research guidelines for submitting code & software for further information.

Data

Policy information about availability of data

All manuscripts must include a data availability statement. This statement should provide the following information, where applicable:

- Accession codes, unique identifiers, or web links for publicly available datasets
- A list of figures that have associated raw data
- A description of any restrictions on data availability

The data that used in this study were obtained from the Alzheimer's disease Neuroimaging Initiative (ADNI) and are available from the ADNI database (adni.loni.usc.edu) upon registration and compliance with the data usage agreement. Data from the BioFINDER sample is available from the authors upon request. Resting-state data of the HCP cohort is freely available online (https://db.humanconnectome.org). A source file for all figures showing individual datapoints can be found in the supplementary.

Field-specific reporting

Please select the one below that is the best fit for your research. If you are not sure, read the appropriate sections before making your selection.

☒ Life sciences ☐ Behavioural & social sciences ☐ Ecological, evolutionary & environmental sciences

For a reference copy of the document with all sections, see nature.com/documents/nr-reporting-summary-flat.pdf

Life sciences study design

All studies must disclose on these points even when the disclosure is negative.

Sample size	We included 81 participants from ADNI phase 3 (ClinicalTrials.gov ID: NCT02854033) based on availability of baseline T1-weighted & resting-state fMRI, 18F-AV45 amyloid-PET and at least two 18F-AV1451 tau-PET visits. The T1-weighted, resting-state fMRI, AV45 amyloid- PET and the first AV1451 image had to be obtained within the same study visit. As an independent validation sample, we included 57 participants from the BioFINDER cohort, that were selected based on availability of amyloid-status, longitudinal AV1451 tau-PET and structural MRI data. To determine a functional connectivity template for the BioFINDER sample, we downloaded spatially normalized (i.e. to MNI space) minimally preprocessed resting-state fMRI images from 500 subjects of the human connectome project (HCP), which are freely available at: https://www.humanconnectome.org
Data exclusions	No data was excluded
Replication	All analyses were replicated across two independent datasets (ADNI & BioFINDER)
Randomization	Allocation on groups was based on diagnosis, so no randomization was performed.
Blinding	Blinding was not possible during analysis

Reporting for specific materials, systems and methods

We require information from authors about some types of materials, experimental systems and methods used in many studies. Here, indicate whether each material, system or method listed is relevant to your study. If you are not sure if a list item applies to your research, read the appropriate section before selecting a response.

Materials & experimental systems

n/a	Involved in the study
☒	☐ Antibodies
☒	☐ Eukaryotic cell lines
☒	☐ Palaeontology
☒	☐ Animals and other organisms
☐	☒ Human research participants
☒	☐ Clinical data

Methods

n/a	Involved in the study
☒	☐ ChIP-seq
☒	☐ Flow cytometry
☐	☒ MRI-based neuroimaging

Human research participants

Policy information about studies involving human research participants

Population characteristics	ADNI: For the Ab- group, we included 28 cognitively normal subjects (CN, MMSE>24, CDR=0, non-depressed). To cover the pre-dementia spectrum of AD, we included 32 CN and 21 mild cognitively impaired subjects (MCI; MMSE>24, CDR=0.5, objective memory-loss on the education adjusted Wechsler Memory Scale II, preserved activities of daily living) with elevated amyloid deposition (i.e. Ab+, global AV45 SUVR > 1.11). Mean tau-PET follow-up time was 1.3 ± 0.52 years in CN Ab- and, 1.27 ± 0.46 years in CN Ab+ and 1.37 ± 0.57 years in MCI Ab+. No significant differences in tau-PET follow up time were found across groups (p=0.817, ANOVA). No significant differences were found between groups in age, gender or education (ANOVA, all p>0.05). As an independent validation sample, we included 57 subjects from the BioFINDER study with available longitudinal AV1451 tau-PET data (table 1). This sample included 16 CN Ab-, 16 CN Ab+, 7 MCI Ab+ and 18 subjects with AD dementia (Ab+). Mean tau-PET follow-up time was 2.03 ± 0.47 years in CN Ab-, 1.91 ± 0.32 years in CN Ab+, 1.82 ± 0.12 years in MCI Ab+ and 1.87 ± 0.34 years in AD dementia. Again, no differences were found in tau-PET follow-up time across groups.
Recruitment	All ADNI subjects were recruited within the Alzheimer's Disease Neuroimaging Initiative (ADNI, see http://adni.loni.usc.edu/). The authors of the study were not involved in subject recruitment. All BioFINDER subjects were recruited at Lund University, Sweden, where the authors were included in subject recruitement.
Ethics oversight	For ADNI, ethical approval was obtained by the ADNI investigators, all participants provided written informed consent. Ethical approval was given by the regional ethics committee at Lund University, Sweden.

Note that full information on the approval of the study protocol must also be provided in the manuscript.

Magnetic resonance imaging

Experimental design

Design type	structural MRI, resting-state fMRI
Design specifications	n/a
Behavioral performance measures	n/a

Acquisition

Imaging type(s)	functional, structural
Field strength	3T
Sequence & imaging parameters	in ADNI, structural MRI was recorded using a 3D T1 weighted MPRAGE sequence with 1mm isotropic voxel-size and a TR=2300ms. For functional MRI, for each subject a total of 200 resting-state fMRI volumes were recorded using a 3D EPI sequence in 3.4mm isotropic voxel resolution with a TR/TE/flip angle=3000/30/90°.<br>In BioFINDER, 1mm isotropic T1-weighted MPRAGE (TR/TE=1900/2.64ms) and Fluid-attenuated inversion recovery (FLAIR; 0.7 x 0.7 x 5 mm3, 23 slices, TR/TE=9000/81ms) MRI images were acquired for all participants on a 3T Siemens Skyra scanner (Siemens Medical Solutions, Erlangen, Germany).
Area of acquisition	whole brain

Diffusion MRI ☐ Used ☒ Not used

Preprocessing

Preprocessing software	FSL, ANTs, AFNI, in-house scripts
Normalization	In ADNI, non-linear spatial normalization parameters were estimated based on structural T1-weighted images using Advanced Normalization Tools (ANTs), to normalize all images to Montreal Neurological Institute (MNI) standard space.<br>In BioFINDER, the MRIs were skull stripped using the combined MPRAGE and FLAIR data, segmented into grey and white matter and normalized to MNI space.
Normalization template	MNI
Noise and artifact removal	For the resting-state fMRI images, we first applied motion correction, regressed out the mean signal from the white matter and cerebrospinal fluid and 6 motion parameters (3 translations & 3 rotations) after which we applied detrending, band-pass filtering (0.01-0.08 Hz) and despiking. To further eliminate motion artifacts, we performed scrubbing, i.e. removal of high- motion frames as defined by exceeding 0.5mm framewise displacement.
Volume censoring	To further eliminate motion artifacts, we performed scrubbing, i.e. high- motion frames as defined by exceeding 0.5mm framewise displacement were replaced with zero-padded volumes.

Statistical modeling & inference

Model type and settings	Univariate
Effect(s) tested	To test our main hypothesis (i.e. association between functional connectivity and covariance in tau change), we applied linear regression, with the vectorized group-average functional connectivity matrix (i.e using ADNI functional connectivity data for the ADNI sample and HCP functional connectivity data for BioFINDER) as a predictor of the vectorized covariance in tau change matrix. For exploratory reasons, we also assessed the association between covariance in tau-PET change and functional connectivity separately for each of the 7 canonical brain networks. The association between whole-brain functional connectivity and covariance in tau-PET change was further determined for ADNI and BioFINDER using the 200 respective shuffled ADNI and HCP functional connectivity null-models, to obtain a null-distribution of the ⍰-values that was used to compare the true ⍰⍰value using an exact test. In ADNI, we further assessed the robustness of the association between functional connectivity and covariance in tau-PET change via bootstrapping. Specifically, we drew 1000 random samples with replacement from the entire group of 53 A⍰+ subjects and assessed for each sample the group-mean functional connectivity, covariance in tau-PET change, as well as the association between them. By saving the 1000 bootstrapping derived ⍰-values we obtained the 95% CI and tested whether the ⍰-value distribution deviated from zero. Note that this bootstrapping approach was exclusively conducted in ADNI, since it required availability of both subject-specific functional connectivity and tau-PET data. The above described whole-brain analyses were further repeated while additionally controlling the regression model for Euclidean distance between each ROI pair, to assess whether associations between functional connectivity and covariance in tau change were independent of distance. Also, we repeated the whole-brain analyses using covariate controlled (i.e. age, gender, education and ApoE4-status) covariance in tau-PET matrices, to ensure that the association between functional connectivity and covariance in tau-PET change was not driven by these covariates.<br>In a next step, we tested whether the level of tau-PET change in a given seed ROI is predictive of the tau-PET changes in

closely connected regions. The rationale is that if tau spreads as a function of functional connectivity, then ROIs with similar tau changes should be connected. To test this, we rank-ordered all ROIs according to their level of tau-PET change. Using linear regression, we tested for each rank-ordered ROI (seed), the group-average functional connectivity to the remaining ROIs (target) as a predictor of the group-average level of tau-PET change in the target ROIs (Figure 5A). Again, we performed the same analyses using the 200 shuffled connectomes, to compare the true □-value with a □-value null-distribution using an exact test. In ADNI, we further determined the robustness of this analysis by repeating the entire procedure using the above described bootstrapping procedure with 1000 randomly drawn samples from the overall pool of 53 A□+ subjects, based on which group-average tau-PET change and functional connectivity were iteratively determined.

Lastly, we tested whether future tau change can be modeled by functional connectivity and tau load at baseline, using three approaches. As a negative control, we tested whether tau spread is a function of baseline tau and the Euclidean distance between ROIs. Specifically, we determined the mean tau-weighted Euclidean distance between a given "tau-receiving" target ROI and all other "tau-seeding" 399 seed ROIs, after multiplying each of the 399 distance values by the respective seed ROIs baseline tau-PET level (Figure 7A, Model 1). For our second approach, we tested whether tau spread can be modeled by combining tau at baseline and functional connectivity. Specifically, we computed the mean functional connectivity between a given "tau-receiving" target ROI and all other "tau-sending" 399 seed ROIs, after multiplying each of the 399 functional connectivity values by the respective seed ROIs baseline tau level (Figure 7A, Model 2). Third, we tested whether adding Euclidean distance as an additional multiplication factor in the above listed model could further improve the association strength with future tau spread (Figure 7A, Model 3).

Within the ADNI and BioFINDER samples, each approach yielded a 400-element vector that was tested as a predictor of annual tau-PET changes in the corresponding 400 ROIs via linear regression. Again, we conducted the same analyses using the shuffled connectomes to compare the true □-value with a □-value null distribution using an exact test. In ADNI, we further performed bootstrapping using 1000 samples based on which group average functional connectivity and tau-PET change were iteratively determined as described above. Within ADNI, the bootstrapped □-value distributions for each of the three approaches were then compared using an ANOVA, to determine which approach yielded the most accurate prediction of longitudinal tau changes.

Next, we assessed prediction model performance on the subject level. For ADNI, we used subject-level tau-PET and functional connectivity data, and for BioFINDER we used subject-level tau-PET and group-level HCP functional connectivity data. Prediction model performance (i.e. □-values reflecting the association between predicted and actual tau-PET changes) was compared across models using an ANCOVA. Here, we also tested whether prediction performance (i.e. model-derived □-values) was associated with age (using linear regression) or with gender and ApoE4 status (using ANOVAs).

Specify type of analysis: [ ] Whole brain [x] ROI-based [ ] Both

Anatomical location(s): Schaefer Atlas, 400-ROI parcellation & 200-ROI parcellation (Schaefer et al., Cereb Cortex, 2018)

Statistic type for inference (See Eklund et al. 2016): n/a

Correction: n/a

Models & analysis

n/a	Involved in the study
[ ]	[x] Functional and/or effective connectivity
[x]	[ ] Graph analysis
[x]	[ ] Multivariate modeling or predictive analysis

Functional and/or effective connectivity: FC was estimated for each subject based on the preprocessed fMRI data from which the mean fMRI time-course was extracted for each of the 400 ROIs by averaging the signal across ROI- specific per volume. Using these 400 ROI-specific timecourses, we assessed functional connectivity as Fisher-z transformed Pearson-moment correlations between all possible ROI pairs. Autocorrelations were set to zero.
